# Complete mitochondrial genome of *Tetraclita squamosa squamosa* (Sessilia: Tetraclitidae) from China and phylogeny within Cirripedia

**DOI:** 10.1080/23802359.2020.1765705

**Published:** 2020-05-20

**Authors:** Meiping Feng, Wenhao Cao, Chunsheng Wang, Shiquan Lin, Dong Sun, Yadong Zhou

**Affiliations:** a Key Laboratory of Marine Ecosystem Dynamics, Second Institute of Oceanography, Ministry of Natural Resources, Hangzhou, China; b Engineering Technology Research Center of Marine Ranching, Shanghai Ocean University, Shanghai, China; c School of Oceanography, Shanghai Jiao Tong University, Shanghai, P. R. China; d College of Marine Ecology and Environment, Shanghai Ocean University, Shanghai, China; e CAS Key Laboratory of Tropical Marine Bio-resources and Ecology, South China Sea Institute of Oceanology, Chinese Academy of Sciences, Guangzhou, China

**Keywords:** *Tetraclita squamosa squamosa*, barnacle, mitochondrial genome, Cirripedia, phylogeny

## Abstract

Here we present the complete mitochondrial genome of *Tetraclita squamosa* *squamosa*, which is 15,191 bp in length with 67.20% AT content. It contains 13 protein-coding genes, 2 ribosomal-RNA genes and 22 transfer-RNA genes. All PCGs except nad4l in *T. squamosa* *squamosa* start with ATN, and terminated with a complete stop codon, except nad3. Phylogenetic analysis based on mitochondrial PCGs shows that *T. squamosa* *squamosa* is clustered with *T. serrata* into a branch (BP = 100). Our result is consistent with previous reports that genus *Tetraclita* and family *Tetraclitidae* are not monophyletic. This study contributes to further phylogenetic analysis within Cirripedia.

The acorn barnacle Tetraclita is a common space occupier in the intertidal zone of tropical and subtropical waters worldwide, and has at least 12 subspecies for the high intraspecific morphological variation (Darwin 1854; Newman and Ross 1976). *Tetraclita squamosa squamosa* (Crustacea: Sessilia: Tetraclitidae) is one of the common major acorn barnacles in Chinese waters (Yan et al. 2012). *Tetraclita squamosa squamosa* was originally described as *Balanus squamosa* by Bruguière 1789, and was separated from another species *Tetraclita japonica* by allozyme electrophoresis and DNA analysis (Yamaguchi 1987; Hasegawa et al. 1996; Chan 2001; Chan et al. 2007). It has been used to monitor the bioavailability of metals in the coastal waters (Rainbow and Phillips 1993; Blackmore 1996, 1999; Blackmore and Chan 1998; Blackmore et al. 1998). Here, we present the first complete mitochondrial genome of the species *T*. *squamosa squamosa*.

Specimens of *T*. *squamosa squamosa* were collected from Daya Bay (114.60°N, 22.55°E) in the South China Sea. The muscle tissue isolated from the fresh specimen was immediately preserved in 95% ethanol and kept in −80 °C in Key Laboratory of Marine Ecosystem and Biogeochemistry, State Oceanic Administration, Second Institute of Oceanography, Ministry of Natural Resources (Barnacle MT-04). DNA was extracted with QIAamp Tissue Kit (QIAGEN, Hilden, Germany) and mitochondrial DNA was amplified with a DNA REPLI-g Mitochondrial DNA Kit (QIAGEN, Hilden, Germany) as directed by the manufacturer. Library construction and sequencing were performed by Biozeron (Biozeron, Shanghai, China) using the Illumina HiSeq 4000 sequencing platform (Illumina, San Diego, CA).

The mitochondrial genome of *T*. *squamosa squamosa* is 15,191 bp in length with a 67.20% AT content (GenBank Accession number: MT232759). It contains 13 protein-coding genes, two ribosomal-RNA genes, and 22 transfer-RNA genes. The length of coding sequences is 10,958 bp (72.13%), and it is shorter than *Tesseropora rosea* which was the lowest among the available mitochondrial genomes of Tetraclitidae in previous report (Cai et al. 2018). Both rRNAs are encoded on the light strand, as in the other crustacean and barnacle mitochondrial genomes. Four PCGs are encoded on the light strand (nd1, nd4, nd4L, and nd5), while the other nine PCGs are located on the heavy strand, which was the same as those of *Tetraclita rufotincta* (Song et al. 2017). Besides two rRNAs and seven tRNAs are encoded on the light strand. Twelve PCGs in *T*. *squamosa squamosa* start with ATN, while nad4l was initiated with GTG. Most of the PCGs terminated with a complete stop codon (TAA or TAG), but one PCG (nad3) had incomplete stop codons (T).

To elucidate phylogenetic relationships of *T*. *squamosa squamosa* with the other barnacles, phylogenetic tree (Figure 1) is constructed based on the PCGs with Maximum Likelihood using phyML ver 3.0 (http://www.atgc-montpellier.fr/phyml/). A total of 30 species with 31 mitochondrial genomes from Cirripedia have been used in the phylogenetic tree (Shen, Chan, et al. 2015, 2016; Shen, Tsang, et al. 2015, 2017; Wares 2015; Shen, Chu, et al. 2016; Shen, Tsoi, et al. 2016; Feng et al. 2019; Ge et al. 2019; Kim et al. 2019). This analysis involved four species belonging to genus *Tetraclita*: *Tetraclita divisa*, *Tetraclita serrata*, *Tetraclita japonica* CN/JP, and *Tetraclita rufotincta*.

**Figure 1.**
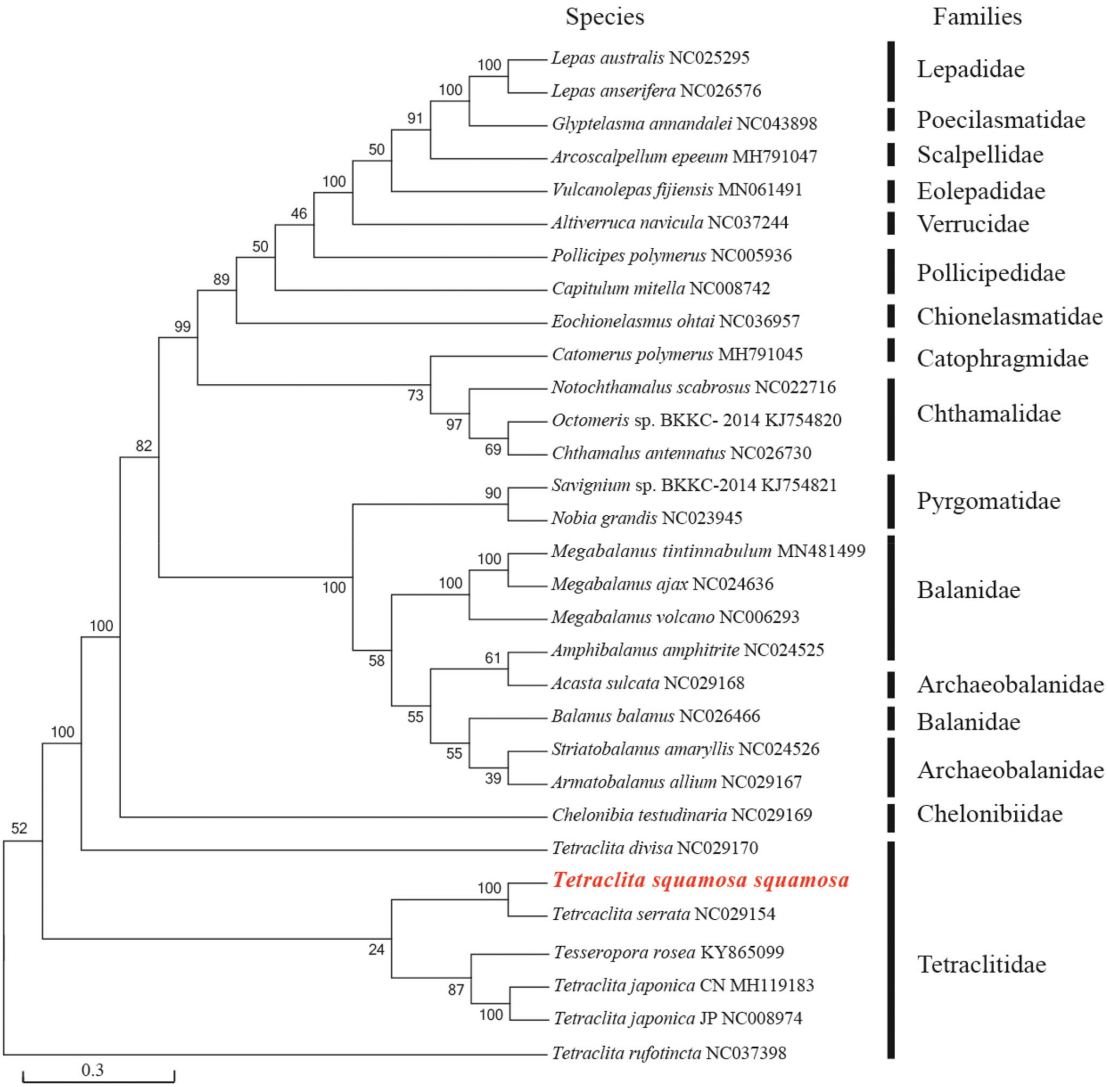
Phylogenetic tree of *Tetraclita squamosa squamosa* and other mitochondrial genomes from Cirripedia based on mitochondrial PCGs.

Result shows that *T. squamosa squamosa* is clustered with *T*. *serrata* into a branch (BP = 100), and the two group with *T. rosea* and *T*. *japonica*, with *T*. *rufotincta* and *T*. *divisa* as the most distantly related species (Figure 1). In the tree, *T*. *divisa* as the most distantly related species within Tetraclitidae, which was consistent with the previous results (Song et al. 2017; Cai et al. 2018). *Chelonbia testudinaria* (Coronulidae) clusters with species from Tetraclitidae, which is consistent with Song et al. (2017). Song et al. (2017) compared genome of four species from family Tetraclitidae, and found the mitochondrial gene order was highly conserved in the family. However, it has been reported that the genus *Tetraclita* does not constitute a monophyletic assemblage (Cai et al. 2018); and Tetraclitidae is not monophyletic (Tsang et al. 2015). Our result is consistent with these previous reports.

In conclusion, we decode the complete mitochondrial genome of *T. squamosa squamosa* and report the phylogenetic analysis for the first time, which will provide data for further molecular and evolutionary analysis within Tetraclitidae and Cirripedia. Further analyses are required to reveal phylogeny and evolution of barnacles.
